# Understanding how best to engage recreationists in biosecurity to reduce the impacts of tree diseases: a review

**DOI:** 10.1042/ETLS20200064

**Published:** 2020-12-01

**Authors:** Clare Hall, Mariella Marzano, Liz O'Brien

**Affiliations:** 1 Forest Research, Northern Research Station, Roslin, Midlothian EH25 9SY, U.K.; 2 Forest Research, Alice Holt Lodge, Farnham Surrey GU10 4LH, U.K.

**Keywords:** biosecurity, engagement, recreationists, tree diseases

## Abstract

Pests and diseases introduced from other countries are known to pose a threat to trees, woods and forests in many locations throughout the world. Once introduced they can be spread inadvertently by countryside visitors hiking, biking or riding along paths and trails through wooded areas. Engaging and communicating with these groups of countryside users to encourage and facilitate positive biosecurity behaviours is crucial for the future resilience of trees, woods and forests. This review draws on literature outlining principles for stakeholder engagement in forestry as well as evaluations of biosecurity campaigns from around the world. Key points are briefly presented for communicators aiming to encourage better biosecurity in the countryside. These include, the need to design campaigns based on the values and motivations of recreationists, using trusted information sources and understanding the importance of combining information with facilities so as to reduce the cost (in terms of effort and inconvenience) to hikers and other countryside users.

## Introduction: the problem of introduced plant pests and pathogens

It is well established that many damaging tree pests and diseases have been accidentally introduced to Great Britain (GB) and elsewhere, such as on plant material and wood packaging imported from other countries [1–3] and through increased global travel [4]. In their native habitats and ecosystems, these invertebrates and pathogens may cause few problems, as they have a natural niche in their established environments and are in balance with other species around them. However, in new environments some of these imported organisms can be fast-spreading and damaging to native and established species and habitats, causing, for example, declines in biodiversity [5], as there are none of the same environmental or biological controls which are found in their native environments elsewhere in the world. For example, populations of the oak processionary moth are controlled by natural predators in countries in southern Europe where the moths are not considered to be a threat, but these predators are not present in northern European countries where the moth is known to defoliate large numbers of oak trees [6]. Recent examples of tree diseases introduced into GB include Dothistroma Needle Blight, Ash Dieback and *Phytophthora ramorum* (*P. ramorum*) [7]. The latter is a threat to the economically important larch tree (*Larix* species) and other species associated with woodlands in GB and has caused widespread damage to forests in the west of the country. It was first detected in GB in 2002 on a viburnum plant at a garden centre in Sussex [8]. *P. ramorum* is an algae-like organism called a water mould and produces extremely high levels of infective spores which can be spread over several miles in mists, air currents, watercourses and rain splash. Where the spores fall to the ground they can be further dispersed in mud and soil on footwear, tyres and animals [9]. Hence recreationists can spread the pathogen as they move through the countryside. This is one example that highlights why it is important to understand how best to support hikers and other recreationists to reduce their potential impact and engage in positive biosecurity behaviours through targeted engagement programmes and communication.

There are other important reasons for engaging with recreationists in relation to biosecurity. Raising awareness and thereby seeking to increase the role of tourists and recreationists in risk mitigation [10] can be a crucial component of the management of countryside areas. Recognising that there is a need for shared responsibility for biosecurity between managers and users of areas, for example with regard to surveillance [11], is also important. This highlights the potentially important role that recreationists can play as citizen scientists for surveillance [12]. Given that mitigation measures might involve path diversions, closure of certain areas, restricted access to water bodies and removal of trees and other vegetation, engaging with recreationists over biosecurity to improve compliance and acceptability is also likely to be key.

## The review

While recognising that there are multiple ways in which recreationists and visitors to countryside areas can contribute to improving biosecurity, the aim of this review is to focus on their role in introducing and spreading pests and pathogens through their recreational activities, and how to help prevent or reduce this through engagement programmes. A key aim is therefore to highlight whether existing behavioural change interventions for biosecurity have achieved the desired changes, and what lessons can be learnt.

To explore these issues the project team reviewed existing literature relating to the following topics:

1. The role of recreationists in the introduction and spread of pests, pathogens, seeds, weeds and other non-native invasive species.
2. Principles of stakeholder communication and engagement for behavioural change.
3. Effectiveness of behavioural change interventions relating to biosecurity.

The literature review focussed on both terrestrial and aquatic environments. The review also extended beyond pests and diseases to include the spread of seeds, weeds and non-native invasive species, due to the paucity of the available evidence relating specifically to recreationists and tree health. Significant evidence and insights may be learnt from the literature on the role of recreationists in the spread of seeds, weeds and non-native species, particularly in the context of their activities, actions, required behaviours, impacts and approaches to messaging and engagement.

To explore the review topics the following keywords were used in multiple combinations in online literature searches using Google, Google Scholar, ScienceDirect and Researchgate (Table 1).

**Table 1.** Key words for the literature search

Headline topics	Secondary topics	Target groups	Actions/activities	Outcome measure
Biosecurity Tree health	Tree diseases Tree pests Invasive species Non-native species Alien species Weeds	Public Stakeholders Tourists/tourism Visitors Recreationists Hikers	Engagement Intervention Programme ‘Keep it clean’ ‘Check, Clean, Dry’	Evaluation

After selecting for relevance the review focused on 10 studies for review topic one (including four reviews), eight studies for review topic two, and seven studies for review topic three.

## The role of recreationists in the spread of plant pests and diseases

The studies reviewed covered activities in a range of terrestrial and aquatic environments, with a focus on forests where possible. They refer to tourists and recreationists, again with a focus on hikers where possible. The studies identified are from North America, Australia, Europe, Antarctica and global reviews.

Countryside areas that are popular with recreationists face many environmental pressures as a result of human activities. The evidence reviewed suggests that recreationists can be instrumental through their outdoor activities in spreading unwanted species that can be damaging to environments such as forests (for a review, see [13]). Also, forests on public land open to recreation have been found to have a higher prevalence of *P. ramorum* than do forests on private lands that are not open for public recreation [14]. Several studies demonstrate the role of recreation and tourism in spreading pathogens [14–18], non-native species [19–21], plant seeds [22] and weeds [17]. Mechanisms by which recreational activities can introduce harmful species or pathogens into forests include footwear, vehicles and bicycle tyres [15]. This applies broadly across ecosystems and is found to be the case in forest, other terrestrial, freshwater and marine environments, including the Antarctic [16,17,21]. For example, hikers are believed to have spread the *P. cinnamomi* spores on boots and camping equipment in some parts of Australia, and to change environmental conditions in ways that increase plant stress [18]. *P. ramorum* has been found to be more common in soil on hiking trails in California than in soil from areas away from trails where the vegetation is undisturbed [14]. Studies have suggested that cleaning boots and equipment before arriving at a countryside destination and again before leaving could reduce the risks of dispersal by recreationists [22]. Overall, the evidence reveals that unintentional pathogen dispersal by recreationists including hikers is likely, thus their engagement in the biosecurity actions such as cleaning boots and other equipment as highlighted by Pickering and Mount [22] is important to protect against future dispersal of tree diseases into new areas.

## Principles of stakeholder engagement for behavioural change

There are many studies that provide guidance and principles relating to stakeholder engagement and interventions designed to facilitate behavioural change. Here, the focus is on a selection of these that have particular relevance to the implementation of biosecurity engagement programmes in woods and forests.

In 2011, guidelines were produced for public engagement in the management of woodlands in the U.K. [23]. The guidelines included some key issues to be addressed before a campaign can be initiated. These include the need to establish the reasons for engaging with people (for example, awareness raising, information provision or behavioural change), and to identify those who have a stake in the programme or those who are the target of it. Communicators should then consider how best to engage each stakeholder or stakeholder group, and identify issues and potential conflicts that might affect engagement. While these guidelines were written within the context of U.K. forest and woodland planning they are widely relevant for other contexts in other countries.

Having clarified the above points, an engagement campaign that aims to encourage biosecurity behaviours should be designed using a number of key principles found in the existing literature. The key engagement principles and communication strategies from the reviewed studies that are of most relevance are presented in Table 2.

**Table 2.** Principles for stakeholder engagement for biosecurity

Points to consider when designing engagement programmes for biosecurity	Specific points from reviewed studies
*Individual values and motivations* Understand the interests, motivations, values and perceptions of those to be engaged. Design engagement programme and communication materials around those motivations, values and perceptions.	Messaging should reflect the diverse contexts and interests of the target audience. Tailor messages for each stakeholder group based on their needs and interests so they can understand, ‘what's in it for me?’. Understand motivations and values and the things people care about. Understand peoples’ risk perceptions. Recognise the importance of peoples’ environmental worldview and views of human nature relationships.
*Social and physical context* Understand the social and physical contexts of those to be engaged. Design engagement programme, communication materials, and necessary infrastructure around those contexts.	Relate to the wider social and physical context of target groups. Social context plays a role in shaping, modifying or driving the individual factors above. Physical context plays a role in behaviour, by prompting or inhibiting certain types of action. For example, how easy is it for hikers to clean their boots in different situations, such as at carparks or hostels, before or after a walk?
*Communication channels used* Understand where and how the target group currently access information relating to related topics such as environmental issues, trees, plants and biodiversity. Design engagement programme around that understanding and utilise multiple communication channels if required.	Adopt a multifaceted approach to communication. Effective public engagement will likely involve multiple methods and will require collaboration and coordination across multiple partners. Identify engagement activities and communication tools that will resonate with the stakeholder group. Communication should be a two-way process of mutual learning between ‘communicators’ and ‘audience’.
*Trusted communicators* Understand which organisations and information sources the target audience trust, rely on and utilise when seeking information on related topics. Investigate feasibility of using these trusted intermediaries in communication and engagement.	Sources providing information and advice need to be trusted by those receiving the information. Channel information through the most appropriate person or organisation that has the respect and trust of the stakeholder group. This might require intermediaries to be involved so these need to be identified. Recognise that more information does not automatically lead to changed behaviours.
*Consistent messaging* Ensure that the engagement programme uses consistent and persistent messaging.	Those seeking to engage the public need messages that are consistent. Engagement processes must have legitimacy with the intended stakeholders.
*Communicate the purpose of actions* Be clear about the positive impacts that the required actions will have.	The aim here should be to explain what people need to do and how to do it. Such explanation should be provided in such a way that it is reasonable to expect people will believe they have the ability to do it. Action should be facilitated if people can see that what they are being asked to do will have a positive impact. People need a sense of personal responsibility, or a feeling of personal obligation to take action.

One study that focused specifically on engaging members of the public in activities related to tree health identified a number of relevant lessons from other sectors [24]. These lessons include the point that messaging should reflect the diverse contexts and interests of the target audience. For example, the likelihood of taking positive biosecurity actions may be related to an individual's environmental worldview or how they view human nature relationships [25]. Thus, communicators need to understand motivations, values, the things people care about and their risk perceptions. This means that messages should be tailored for each stakeholder group based on their needs and interests so they can understand, ‘what's in it for me?’.

Key principles have been developed that could be used to guide forestry interventions aimed at achieving behavioural change [26]. These principles were developed for diverse forestry behaviours ranging from activities such as felling and timber harvesting to social, recreational and cultural activities conducted in a woodland or forest setting. They could also include biosecurity behaviours. The study considered interventions in other sectors including health, energy and transport that focused on behaviour and behaviour change, to look for transferable lessons for the forestry sector. These included the need to relate to the wider social and physical context of target groups as social context plays a role in shaping, modifying or driving the individual factors described above. It also recognised that physical context plays a role in behaviour, by prompting or inhibiting certain types of action. For example, countryside managers should consider how easy it is for hikers to clean their boots in different situations, such as at carparks, hostels or train stations before setting off on a walk or before heading home. Of relevance to this point, a study carried out in Scotland, U.K., in 2018 found that key local businesses and tourist organisations expressed a willingness to provide the necessary biosecurity equipment for hikers, such as brushes, boot scrapers and taps [27].

An Australian project called ‘Engaging in Biosecurity’ developed guidelines for engaging communities in biosecurity for agriculture [28]. The project aimed to develop an engagement framework that identified what enables and hinders effective community engagement about biosecurity issues. This was done by profiling six existing biosecurity engagement programmes and conducting four biosecurity engagement trials. A number of principles that address the enablers and barriers identified through their work are also included in Table 2. These principles include the fact that sources providing information and advice need to be trusted by those receiving the information, thus information needs to be channelled through the most appropriate person or organisation that has the respect and trust of the stakeholder group. This might require intermediaries to be involved so these need to be identified. Research with hikers in the Loch Lomond National Park in Scotland revealed key trusted organisations and communication media that were relied upon and commonly utilised. Specifically, people said they get information online about the environment, plants and trees, and conservation organisations were mentioned as trusted sources they use to find out about trees and plants [27]. Nevertheless, it is important to recognise that providing additional information, even through trusted sources, and well-used channels, does not automatically translate into additional knowledge and awareness, and changed behaviours [29]. Communication should also be a two-way process of mutual learning, such that communicators understand the recreationists’ perspectives on risks, their observations, concerns and experiences [30]. Some further lessons described by authors include the need to adopt a multifaceted approach to engagement at various scales, thus effective public engagement will likely involve multiple methods and will require collaboration and coordination across multiple partners [28].

A review of literature relating to engaging people in biodiversity issues identified a number of factors which could explain different levels of engagement, and that could therefore be relevant when designing engagement programmes for biosecurity issues [31]. Again, some of these factors feature in Table 2. These include the need to make clear what people need to do, and how. These ‘action’ messages should be provided in such a way that it is reasonable to expect people will believe they have the ability to carry out the required actions. For example, messages need to be clear and straightforward. It is also important to explain how the actions required will have a positive impact, such that people may feel a sense of personal responsibility, or personal obligation to take action.

## Effectiveness of behavioural change interventions relating to biosecurity

There is very little evidence about how best to engage with recreationists about tree health and plant biosecurity in order to increase awareness and change behaviours. This section draws on seven studies that have reported evaluations of biosecurity engagement and education campaigns in the U.S.A., Australia, New Zealand and the U.K. Two studies focussed on programmes aimed at preventing the movement of firewood by U.S.A. campers to stop the spread of tree borers [32,33]. Another study with recreationists in Australia explored an education programme concerning the spread of a root rotting fungus [34]. Recognition and awareness of a biosecurity campaign logo and slogan used in GB was investigated with regard to *P. ramorum* [27]. There is also evaluation of programmes relating to alien invasive species in aquatic environments, including the New Zealand version of the ‘Check, Clean, Dry’ campaign aimed at water users [35], and two studies examining outreach programmes in Illinois, U.S.A. [25,29].

These evaluation studies present a mixed picture in terms of the effectiveness of biosecurity engagement campaigns aimed at raising awareness and encouraging new behaviours among recreationists. Importantly, simply being exposed to information about a biosecurity issue did not translate into large numbers of people gaining new knowledge and changing behaviours [24,29]. Familiarity with outreach campaign logos has generally been found to be low, with recreationists often not understanding slogans [25,27].

Two examples of campaigns that achieved some success in terms of behavioural change of recreationists both combine the provision of information with practical resources and biosecurity equipment. Campaigns aimed at U.S.A. campers regarding the movement of firewood seem to have had some success at reducing the numbers of campers transporting firewood from places outside of campsites through understanding their motivations and behaviours [32,33]. This program also made it easier for campers to purchase firewood on-site on their arrival, thus reducing the cost (in terms of effort) to recreationists. The ‘Check, Clean, Dry’ campaign in New Zealand [35] is another example of a biosecurity engagement programme that has been found to be successful. Again, the success of this programme lies in the fact that it combined information provision to raise awareness with the provision of practical resources such as cleaning stations, detergent sachets and spray bottles for recreationists.

Overall, looking at evaluations of biosecurity engagement campaigns tells two stories. On the one hand, a number of campaigns in different countries in both terrestrial and aquatic environments have not succeeded in raising awareness and changing self-reported behaviours, even when people have been exposed to information. On the other hand, U.S.A. campaigns aimed at campers to stop the movement of firewood report some positive achievements, and the ‘Check, Clean, Dry’ campaign in New Zealand appears to have increased awareness and engagement with positive biosecurity actions by water-based recreationists. A number of conclusions from the ‘Check, Clean, Dry’ evaluations about its strengths and reasons for success, are described here as they have relevance for all biosecurity engagement campaigns, including those for woods and forests. The campaign used multiple different communications channels with a focus on signs at key recreational sites, and cleaning stations were also provided at high-risk sites. Biosecurity messaging about actions to take were simple and consistent across sites. Evaluation of engagement programmes was key to understanding what worked and what did not work.

## Summary

- Recreationists, including hikers, are known to contribute through their activities in the countryside to the spread and dispersal of invasive pests and diseases, including in woodland and forests. Thus, understanding how best to engage and communicate with such groups to encourage positive biosecurity behaviours is crucial for resilient treescapes. More broadly, for those concerned about biosecurity, recreationists and visitors to countryside areas can play an important role, not only in helping to avoid the introduction and spread of pests and pathogens, but also in ongoing risk mitigation and surveillance.
- Principles for stakeholder engagement to encourage behavioural change emphasise the need to design engagement campaigns using the values and motivations of importance to the target group in order to effectively frame the issue. Further research is needed into the values and attitudes of hikers towards the environment, trees, diseases and good practice in the countryside; this will help to inform the design of biosecurity campaigns in GB and elsewhere. Linked to this point, engagement campaigns should place more emphasis on information exchange, learning from recreationists about their own experiences, concerns and observations. This reflects IPPC guidance on Pest Risk Communication which stresses that ‘*pest risk communication is used to enable mutual understanding and dialogue among all stakeholders about plant health issues*’ ([30], P5).
- Communicators need to use trusted information sources for communicating with hikers and other recreationists about biosecurity. It is well established that using trusted sources for communicating with the public about environmental challenges is important [36]. It is important to note that information on its own is unlikely to lead to behaviour change [37]. Successful biosecurity campaigns combined equipment with information provision.
- Thus far, there has been a lack of evaluation of outreach and engagement programmes designed to encourage behaviour change. There is an urgent need to increase understanding of the effectiveness of such campaigns by combining on the ground activities with before and after studies of behaviours, ideally observed rather than self-reported. Further, principles of stakeholder engagement as described in many publications should be applied and tested with regard to biosecurity engagement campaigns.

## Abbreviations

GB: Great Britain
